# The role of CXCR3 and its ligands in cancer

**DOI:** 10.3389/fonc.2022.1022688

**Published:** 2022-11-21

**Authors:** Xiaoming Wang, Yangyang Zhang, Sen Wang, Hongyan Ni, Peng Zhao, Guangyu Chen, Benling Xu, Long Yuan

**Affiliations:** ^1^ Department of Surgery, The Affiliated Cancer Hospital of Zhengzhou University & Henan Cancer Hospital, Zhengzhou, China; ^2^ Department of Surgery, Henan No.3 Provincial People’s Hospital, Zhengzhou, China; ^3^ Department of Immunotherapy, The Affiliated Cancer Hospital of Zhengzhou University & Henan Cancer Hospital, Zhengzhou, China

**Keywords:** CXCR3, CXCR3 and its ligands, CXCL9, CXCL10, CXCL11

## Abstract

Chemokines are a class of small cytokines or signaling proteins that are secreted by cells. Owing to their ability to induce directional chemotaxis of nearby responding cells, they are called chemotactic cytokines. Chemokines and chemokine receptors have now been shown to influence many cellular functions, including survival, adhesion, invasion, and proliferation, and regulate chemokine levels. Most malignant tumors express one or more chemokine receptors. The CXC subgroup of chemokine receptors, CXCR3, is mainly expressed on the surface of activated T cells, B cells, and natural killer cells, and plays an essential role in infection, autoimmune diseases, and tumor immunity by binding to specific receptors on target cell membranes to induce targeted migration and immune responses. It is vital to treat infections, autoimmune diseases, and tumors. CXCR3 and its ligands, CXCL9, CXCL10, and CXCL11, are closely associated with the development and progression of many tumors. With the elucidation of its mechanism of action, CXCR3 is expected to become a new indicator for evaluating the prognosis of patients with tumors and a new target for clinical tumor immunotherapy. This article reviews the significance and mechanism of action of the chemokine receptor CXCR3 and its specific ligands in tumor development.

## Introduction

Chemokines are chemotactic cytokines that bind heparin, a class of small-molecule proteins that interact by activating a subpopulation of seven transmembrane G protein-coupled chemokine receptors. The target cell specificity of each chemokine is determined by its cognate receptor expression pattern. Two major subfamilies (CX3C and CXC) and two minor subfamilies (CC and C) can be classified according to the position of the two conserved cysteine residues at the N terminus (1, 2). Chemokines bind to a family of 18 G protein-coupled receptors that mediate intracellular signaling. The physiological function of these receptor or ligand pairs is to direct the migration of leukocytes expressing a subset of the receptors to tissue injury or inflammation sites and to participate in the homeostasis of the internal tissue environment. The first chemokine was discovered by Walz et al. in 1977 (3). This chemokine is a procoagulant and vasopressor factor known as platelet factor 4 (CXCL4). Chemotactic functions were not attributed to this growing family of ligand until the discovery of the chemotactic properties of interleukin 8 (IL-8), also known as CXCL8, in neutrophils, when chemokine-induced cellular chemotaxis was appreciated. The standard term “chemokines” was coined at the 3rd International Symposium on Chemokines in Baden in 1992. More than half of the known chemokine receptors are considered determinants of the malignant behavior of tumors. Chemokines can bind to and mediate the function of target cells expressing their corresponding receptors in the immune environment, thereby exerting immune function in the body (4). Chemokines play an essential role in the complex tumor microenvironment (TME), in which the host and cancer cells release a series of chemokines that lead to the recruitment and activation of various cells to induce a tumor-promoting or anti-tumor response. Chemokines mediate the directional movement of cells in the TME and have a variety of regulatory functions, which can not only act on immune cells but also directly act on tumor cells, thus playing a complex biological role. CXCR3 (C-X-C motif chemokine receptor 3) participates in tumorigenesis and promotes the formation of tumor-associated blood vessels by binding to its cognate CXC chemokine ligand 9(CXCL9)/10/11. It also mediates the infiltration of immune cells into tumor tissues. This dual anti-tumor and pro-tumor effect seems to be closely related to CXCR3 variants (CXCR3-A, CXCR3-B, and CXCR3-alt), which play different roles. This review focuses on CXCR3 and highlights the role of CXCR3 and its ligands in cancer progression.

## Composition and role of CXCR3 and its ligands

**Figure 1:**
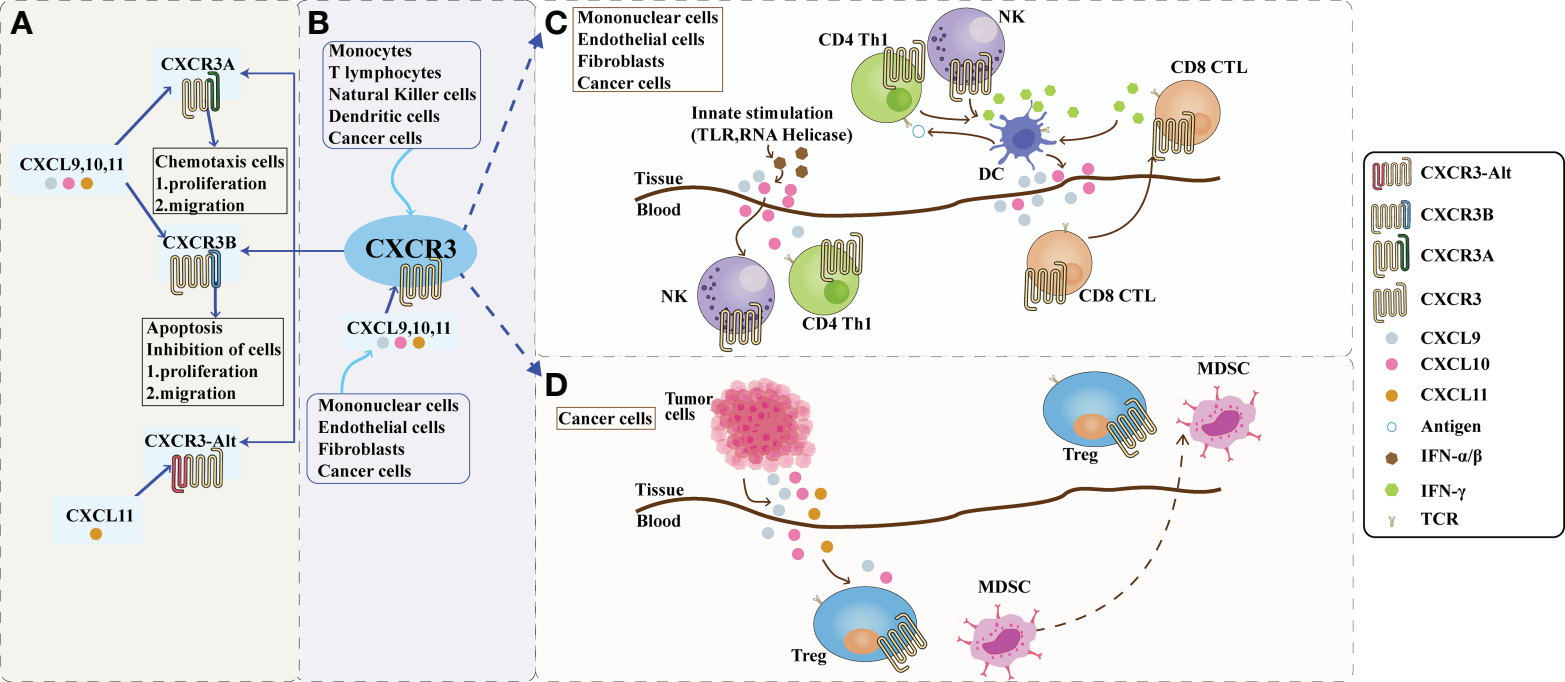
The role of CXCR3 and its ligand pathway in the tumor microenvironment. **(A)**: Composition of CXCR3. Depending on the piece of the amino terminus of the receptor, CXCR3 can be divided into three splice variants: CXCR3-A, CXCR3-B, and a truncated variant CXCR3-alt. CXCR3-A and CXCR3-B have ligands CXCL9, CXCL10, and CXCL11, while CXCR3-alt binds only CXCL11. CXCR3A promotes cell proliferation and migration in the tumor microenvironment, while CXCR3B inhibits cell proliferation and migration. **(B)**: The CXCL9,10,11/CXCR3 axis in the tumor microenvironment. CXCL9, 10, and 11 are mainly secreted by monocytes, endothelial cells, fibroblasts, and cancer cells in response to IFN-γ. The CXCL9,10,11/CXCR3 axis works in two main directions; paracrine signals for immune activation and autocrine signals for cancer cell proliferation and metastasis. **(C)**: Paracrine secretion of CXCR3 is also known as innate immune activation. This axis is primarily used for migration, differentiation, and activation of immune cells for paracrine signaling. Immunoreactivity occurs through this axis by recruiting CTLs, NK cells, and macrophages. Activating TLRs and RNA helicases leads to the release of IFN-a/β from histiocytes and endothelial cells, followed by the secretion of chemokines such as CXCL10; CXCL10 recruits NK and CD4+ Th1 cells into the target tissue, while CD4+ Th1 cells release IFN-γ in an antigen-specific manner. DCs and other resident cells in the tissue take up IFN-γ and induce the production and secretion of CXCL9 and CXCL10; secreted CXCL9 and CXCL10 recruit CD8+ CTL into the tissue. IFN-γ produced by CD8+ CTL further stimulates tissue cells to produce more CXCL9 and CXCLI0. Increased chemokine release and inflammatory responses are amplified, leading to further recruitment of CXCR3-expressing Th1 and CD8+ TIL cells. Figure **(D)**: The autocrine function of CXCR3. In terms of autocrine signaling, cancer cells are predisposed to metastasis due to the activity of tumor-derived ligands, mainly through CXCR3A. Tumor-derived chemokines are also responsible for the recruitment of Th cells, Tregs, and MDSCs, which play a role in creating a tumor-friendly microenvironment. CTLs, cytotoxic lymphocytes; NK, natural killer cells; MDSCs, myeloid-derived suppressor cells; Th1, T helper 1; Tregs, regulatory T cells.

The chemokine receptor CXCR3 is induced by three interferon-γ (IFN-γ)-inducible ligands, CXCL9 (C-X-C motif chemokine ligand 9), CXCL10 (C-X-C motif chemokine ligand 10), and CXCL11 (C-X-C motif chemokine ligand 11) activation (5). It is a seven-transmembrane G protein-coupled receptor encoding 368 amino acids, with a relative molecular mass of approximately 41 kDa and a full-length cDNA of 1104 bp (6). Depending on the composition of the amino-terminus of the receptor, CXCR3 can be divided into three splice variants: CXCR3-A, CXCR3-B, and a truncated variant, CXCR3-alt. CXCR3 was first cloned in 1996 (7), and two splice variants, CXCR3-A and CXCR3-B, were subsequently identified. CXCR3-alt was first reported in 2004, in which Ehlert identified a variant human chemokine receptor, CXCR3, which was generated by alternative splicing by skipping exons. It was experimentally observed that RNA processing requires a variant C-terminal protein sequence with a specific transmembrane structure that is distinct from all known functional chemokine receptors. Therefore, this splice variant is referred to as the CXCR3-alt (8). The ligands of CXCR3-A and CXCR3-B are CXCL9, CXCL10, and CXCL11, whereas CXCR3-alt binds only to CXCL11 (9). Among these, CXCR3-A and CXCR3-B are the two most studied isomers (10). CXCR3-A is mainly expressed in activated T lymphocytes and natural killer (NK) cells (11, 12). At the same time, CXCR3-B is primarily distributed on vascular endothelial cells, while relatively little research has been conducted on CXCR3-alt, which is currently believed to exert biological effects mainly in combination with interferon-induced T cell alpha chemoattractant (I-TAC). For the study of CXCR3 and its ligands, in recent years, many studies have verified that the expression of CXCR3 and its ligands in the body is closely related to tumor immunity, tumorigenesis, and metastasis (13–19). Chemokines not only coordinate the migration of immune cells but also play an essential role in the maturation of immune cells and the generation of adaptive immune responses (20). Dysregulation of chemokines can cause various human diseases, including autoimmune and inflammatory diseases. CXCR3 and its ligands are shown in **Figure 1A**.

CXCR3 is expressed not only in activated T cells but also in small amounts on some epithelial cells and endothelial cells; almost no expression has been observed in quiescent T cells and monocytes. CXCR3 plays a crucial role in migrating effector T cells to inflammation and tumor sites (21, 22). CXCR3 activation preferentially results in the high expression of Th1-type CD4+ T lymphocytes, effector CD8+ T lymphocytes, NK cells, and NKT cells. CXCR3-A is expressed on Th1 T lymphocytes, cytotoxic CD8+ T lymphocytes, and activated B and NK cells and mediates the targeted migration of these cells to inflamed lymph node reactive sites. CXCR3-B is an alternatively spliced variant containing a longer NH2-terminal extracellular structural domain that is predominantly distributed in vascular endothelial cells. CXCR3 and their receptors are distributed in different body tissues, and their expression can regulate the directional migration of different immune cells (23). It can significantly reduce endothelial cell DNA synthesis and promote apoptosis of the vascular endothelium, thereby inhibiting angiogenesis (24). Transfection of CXCR3-A or CXCR3-B into human microvascular endothelial cells revealed that CXCR3-B inhibits DNA synthesis and induces apoptosis, whereas CXCR3-A supports cell survival and further chemotaxis. CXCR3-alt exerts its biological effects mainly in combination with I-TAC, and despite the apparent similarities with CXCR3-B, its specific role is still controversial (8).

**Figure 2:**
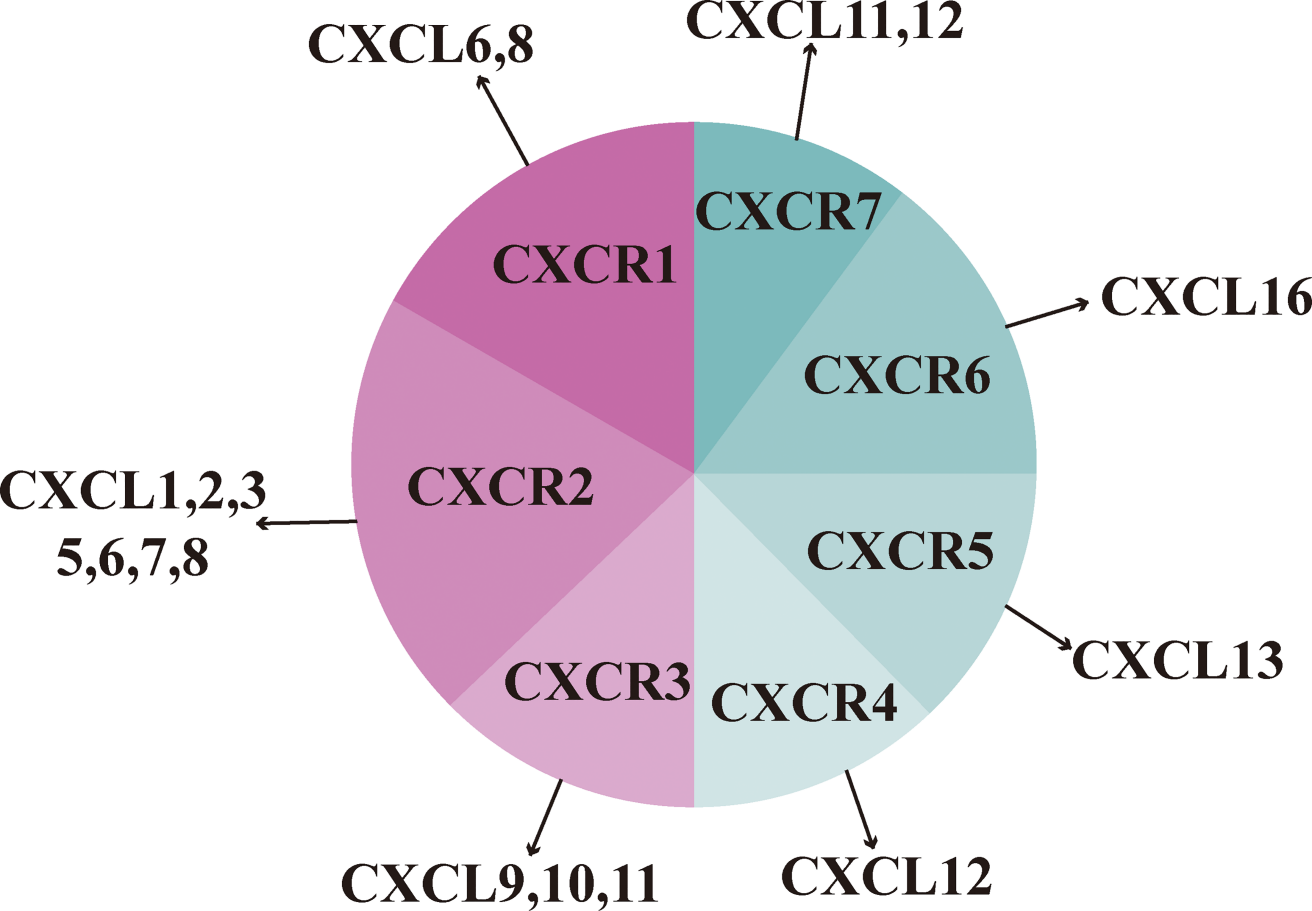
CXC chemokines with their cognate receptors.

CXC chemokines are classified into two forms according to the presence or absence of glutamate-leucine-arginine (ELR) motifs: those with ELR motifs promote neutrophil migration and angiogenesis and those without ELR motifs promote lymphocyte migration and inhibit angiogenesis (25). CXCL9, 10, and 11 are ELR-negative CXC chemokines that can exert an inhibitory effect on tumor growth by inhibiting angiogenesis and thus tumor growth. However, it has also been suggested that it promotes tumor proliferation and metastasis, possibly because the ligand exerts different effects on different CXCR3 variants (26). CXCL9, 10, and 11 mainly regulate immune cell activation, differentiation, and migration in the CXCR3 pathway and exert immune responses by recruiting immune cells, such as cytotoxic lymphocytes (CTL), NK cells, NKT cells, and macrophages (27). In terms of immune function, CXCL9 and CXCL10 can enhance the effector function of Th1 cells; however, CXCL11 binds to different sites on the CXCR3 receptor and mediates opposite effector functions, promoting the expression of differentiated Foxp3-regulatory T cells (T-regulatory 1, Tr1), which in turn inhibits the function of effector T cells (28, 29). CXC chemokines with their cognate receptors are shown in **Figure 2**.

In the TME, the migration and activation of immune cells are closely related to the chemokine family. Among them, CXCL9 and the corresponding chemokine receptor CXCR3 play crucial roles (5, 30, 31). CXCL9, also known as IFN-γ-induced mononuclear factor (MIG), plays a vital role in the chemotaxis of immune cells. CXCL9 can be secreted by a variety of cells, including immune cells (T lymphocytes, NK cells, dendritic cells, macrophages, and eosinophils) and non-immune cells (endothelial cells, tumor cells, and fibroblasts) (32). CXCL9 mediates lymphocyte infiltration to focal sites and inhibits tumor growth. Expression of chemokine CXCL9 is closely associated with immune cell migration, differentiation, and activation in the TME. It positively affects the anti-tumor immune response of CXCR3-expressing CD8+ T lymphocytes, Th1 cells, cytotoxic T lymphocytes, and NK cells (33–35). CXCL9 is expressed at low levels in monocytes, endothelial cells, and fibroblasts during TME homeostasis. However, its expression is significantly upregulated in response to cytokine stimulation such as IFN-γ and tumor necrosis factor-α (TNF-α) (5, 31, 36). It has been suggested (33) that tumor cells with defective CXCL9 expression have greater tumorigenicity than CXCL9-expressing tumor cells in mouse experiments, followed by further speculation that CXCL9 expression deficiency is a mechanism by which tumor cells evade anti-tumor immune responses, leading to immune escape. Further analysis of the TME revealed that CXCL9 expression was upregulated by IFN-γ induction and could bind to CXCR3 expressed by T, B, and NK cells to recruit lymphocytes to the TME (30, 37). At the same time, many experts have discovered that CXCL9 under-expression significantly impairs the immune cell-mediated anti-tumor immune response (34, 35, 38). Thus, high CXCL9 expression is associated with prolonged overall survival in various malignancies, such as kidney cancer and pancreatic ductal adenocarcinoma. Some experts used immunohistochemical techniques to detect CXCL9 expression in kidney cancer tissues and found that CXCL9 expression was negatively correlated with tumor size and microvessel density, and positively correlated with the number of CD8+ T cells infiltrating the tumor (39, 40).

Chemokines and their receptors play an essential role in the development of tumorigenesis. CXCL10 is a chemokine induced by IFN-γ, and CXCR3 is the only receptor for the CXCL10 ligand. Recent studies have shown that the CXCL10/CXCR3 signaling pathway is closely associated with inflammation and tumorigenesis (41). Another name for chemokine CXCL10 is IFN-γ-inducible protein 10 (IP10). Human CXCL10 was first identified as an early response ligand induced by IFN-γ in a lymphoma cell line (U937). CXCL10 is mainly expressed by various cells, including fibroblasts, endothelial cells, hepatic parenchymal cells, and keratinocytes. CXCL10 is expressed at low levels in the thymus, spleen, and lymph node stroma and can be induced by IFN-α, IFN-β, IFN-γ, or LPS stimulation. It is highly expressed in a variety of cells, including endothelial cells, fibroblasts, monocytes, and neutrophils, among others, and is a chemoattractant for activated T cells (42). CXCL10, in addition to inducing effector Th1 cells, also recruits CXCR3+CD8+ T cells to tumor sites and induces granzyme-b production through these cells, thereby enhancing the anti-tumor effect (43). It has been shown that the CXCL10 ligand can be involved in the development and progression of various inflammatory diseases and tumors by recruiting T cells, B cells, monocytes/macrophages, and NK cells that express the CXCR3 ligand. CXCL10 is highly expressed in gastric, breast, lung, and multiple osteosarcoma tissues, and is closely associated with the malignant phenotype of these tumors (30). The CXCL10 ligand is also a vasopressor that recruits anti-tumor T lymphocytes for potential anti-tumor effects, but autocrine CXCL10 signaling by tumor cells can also promote tumor cell proliferation, angiogenesis, and metastasis. The literature has indicated that the CXCR3 ligand is aberrantly expressed in various tumors, such as breast, myeloma, and colon cancer, and is associated with tumor metastasis and prognosis (44).

The chemokine CXCL11, also known as I-TAC or IFN-γ-inducible protein 9 (IP-9), is induced by IFN-γ, IFN-β, and IFN-α, with weak induction of IFN-α (30). CXCL11 is the ligand that binds with the highest affinity to CXCR3, and this interaction promotes eosinophil granulocyte release from the bone marrow and peripheral tissues (2). CXCL11 participates in the inflammatory response by selectively recruiting activated T cells to the sites of inflammation. In addition, CXCL11 binds to CXCR7, which is associated with cell invasiveness and reduced apoptosis of tumor cells (45). CXCL11 can exert its biological functions through CXCR3-mediated activation of signaling pathways (9). Palladino et al. (46) found that the N-terminal structural domain of CXCL11 contains CXCR3 recognition and binding sites, further elucidating CXCR3 as a critical receptor for CXCL11 from a protein structural perspective. Recent studies have shown that CXCR3 expression is significantly elevated in gallbladder cancer tissues and is closely associated with tumor metastasis and prognosis (47).

## The role of CXCR3 and its ligands in immunity

### CXCR3 expression on T cells

CXCR3 is induced after naive T cell activation and is highly expressed on effector CD8+ T cells and Th1-type CD4+ T cells (37). CXCR3 and its three chemokine ligands, CXCL9, CXCL10, and CXCL11, play a central role in regulating effector T cell homing. Single-cell RNA sequencing analysis in skin of cutaneous T-cell lymphoma patients confirmed that CXCL9 and CXCL11 were primarily macrophage-derived and that skin T-cells expressed CXCR3 (48). CXCL9 and CXCL11 recruit CXCR3+ T cells to the skin and overexpressed in rashes, higher CXCL9 and CXCL11 expression in macrophages had more CD8+ T cells in the tumor microenvironment. Emerging data suggest that TAM-mediated expression of the chemokine CXCL9 regulates the recruitment and localization of CXCR3-expressing stem-like CD8+ T cells, which underlies the clinical response to anti-PD-L1 therapy (49). Torphy et al. found that ablation of orphan G protein-coupled receptor 182 (GPR182) leads to increased intratumoral concentrations of multiple chemokines, which triggered a CXCR3-dependent increase in effector CD4+ and CD8+ T cell infiltration, and thereby sensitizes poorly immunogenic tumors to immune checkpoint blockade and adoptive cellular therapies. CXCR3 blockade reverses the improved anti-tumor immunity and T-cell infiltration characteristics in GPR182-deficient mice. GPR182 identified as an upstream regulator of the CXCL9/CXCL10/CXCR3 axis that limits antitumor immunity and as a potential therapeutic target for immuno-cold tumors (50).

Glioblastoma is the most common and aggressive type of tumor among primary brain cancers. Kollis (51) et al. validated chemokine receptors and integrins at the protein level using single-cell RNA sequencing (scRna-Seq) and flow cytometry of biopsy specimens from patients with glioblastoma. They confirmed that the receptors CCR2, CCR5, CXCR3, CXCR4, CXCR6, CD49a, and CD49d were more abundant in the glioblastoma-infiltrated T cell population compared with peripheral blood. CXCR3 can mediate the entry of T cells into the brain parenchyma, providing an essential basis for PD-1 treatment.

CXCR3 expression in peripheral T cells can be used as a biomarker to predict the efficacy of PD-1 antibodies. In the TME of a mouse melanoma model, exogenous supplementation of CXCL9/10 can assist PD-1 antibody in inhibiting tumor growth by increasing the proportion of CXCR3+ T cells (52). Furthermore, many other studies have suggested that intratumoural CXCL10 signaling is positively correlated with the efficacy of immune checkpoint blockade (ICB) treatment (53, 54). Li et al. (55) demonstrated the combined effect of the CXCL10 ligand and PD-1 antibody in a mouse colon cancer model by inserting the CXCL10 ligand into the oncolytic adenovirus binding them to the PD-1 antibody. Oncolytic adenovirus (AdvCXCL10) enhances PD-1 antibodies by increasing the number of CXCR3+ T cells in the TME. This effect was lost when CXCR3 signaling was blocked. Studies have shown that the CXCR3-CXCL9/10 axis is crucial in combined immunotherapy (53, 56, 57). The previous study by Chow (36) and the CXCR3 blocking experiment in the study by Han (52) both confirmed that the anti-tumor effect of PD-1 blockade therapy was significantly weaken after the loss of CXCR3 signaling. Liu (58) found that the combination therapy induced antigen-specific CD8 T cell populations with active chemokine signaling (CXCR3+/CCL5+), lower co-inhibitory receptor expression (Lag3-/Havcr2-), and higher cytotoxicity (FasL +/Gzma +) by single-cell sequencing. CXCR3 is upregulated in neoantigen-specific T cells. Blocking T cell egress from the lymph nodes by FTY720 (an S1P receptor agonist to block T cell egress from the lymph nodes) significantly inhibited the efficacy of combined therapy. Thus, it demonstrated that neoantigen-specific T-cell infiltration maybe through the CXCL9-CXCR3 pathway. PD-(L)1 blockade synergized with OX40/4-­1BB costimulation by dramatically enhancing stem-­like TIL presence *via* a CXCR3-­dependent mechanism (59). Efficacy was largely dependent on CXCR3-mediated intratumoral recruitment and trafficking of stem-cell CD8+ T cells recruited from the periphery (36, 49, 60, 61). Recent studies also show that CXCL9 positively correlates with CD8+ TIL frequency across various human cancers and the response rate of patients with melanoma to anti-PD­1 (62–64). CXCR3 may play a role in stem cell-like CD8+ T cells locating the microenvironment, CD8+ T cells can acquire effector cell-like functions by appropriate activation stimuli. Finally, CXCR3 ligands can directly induce the activation of stem-like CD8+T cells (65).

### CXCR3 expression on NK cells

Natural killer cells can recognize cancer cells independently of antigen presentation based on MHC I (self and non-self). They are an essential link between innate and adaptive immune responses. NK cells express a variety of ligands of the checkpoint family, such as PD-1, TIGIT, TIM-3, LAG3, CD96, IL1R8, and NKG2A. TME can also shape NKs, converting them into a pro-tumoral, pro-angiogenic “nurturing” phenotype through “decidualization.” The features of these NKs include expression of CD56, CD9, CD49a, and CXCR3; low CD16; and poor cytotoxicity. NK cells have cytotoxic and regulatory activities. They induce apoptosis and death by releasing perforin and granzyme, on the other hand, they coordinate the innate response by secreting soluble immunomodulatory factors, such as cytokines and chemokines. These factors act on hematopoietic cell recruitment and activation (66). Due to the duality between innate and acquired immunity of NK cells, TME may profoundly affect their function of contrasting or supporting tumor growth and promoting immune escape. Studies have confirmed that TME can directly promote blood flow recruitment and CD56+CD16-NK accumulation at tumor sites by promoting the switch of chemokine expression in patients with non-small cell lung cancer. CD56+ NK cells correlated with the downregulation of CXCL2 and overexpression of chemokines (CD56 NK cells were preferentially attracted), CXCL9, CXCL10, and CCL19. These chemokines promote low cytotoxic CD56+ NK recruitment by binding to CXCR3, ultimately leading to tumor escape (67). NK cells have a variety of functions. One of the subtypes of NK Cells is Decidual Natural Killer Cells, it was first identified in the Decidual placenta and uterus, hence named dNKs (68). When added to tumor cell xenografts, dNK cells can stimulate neovascularization and tumor growth. CXCR3 is another marker of dNK and expressed in tumor infiltrating -NK cells of colorectal cancer, breast cancer, melanoma, and glioblastoma (69, 70). Fitzgerald et al. confirmed that the CXCR3+ cell population was composed of about 1/3 CD4+ T cells, 1/3 CD8+ T cells, and 1/3 NK cells by collecting spleen cells from C57BL/6 mice (71). They propose that Dipeptidyl peptidase (DPP4) inhibition increases tumor content of CXCL9/10, which recruits CXCR3 + NK and T cells, and DPP8/9 inhibition activates the inflammasome, resulting in proinflammatory cytokine release and Th1 response, further enhancing the CXCL9/10-­CXCR3 axis. However, either mechanism enhance the response of CXCR3 for anti-tumor immune response.

### CXCR3 expression on hypertrophic and macrophages

Fletcher et al. found that the chemokine CXCL10 is up-regulated in neurofibromatosis type I mice that develop neurofibromas to some extent (72). In these neurofibroma-prone mice, global deletion of the CXCL10 receptor CXCR3 prevented neurofibroma development, and anti-CXCR3 antibodies reduced tumor numbers to some extent. CXCR3 expression in inflamed nerves and neurofibromas is localize to T cells and DC. CXCR3 expression is required to maintain elevated macrophage numbers in neurofibromatosis type I mutant nerves. CXCR3 deletion does not prevent mast cell recruitment but results in a delayed resolution of macrophage infiltration. Loss of the CXCR3 receptor is sufficient to prevent neurofibroma development and ultimately reduce neuropathology. Xie et al. found that with the increase in respiratory mechanical energy, the more serious the lung injury in rats and patients, the higher the expression of CXCL10/CXCR3 in blood (73). Their research also showed that CXCL10 regulated the migration of mast cells to inflammatory sites. They speculated that CXCL10/CXCR3 maybe participate in mechanical lung injury by mediating mast cell chemotaxis. Previous studies showed that activating specific chemokines enhances mast cell migration (74, 75). Furthermore, CXCL10 and CXCR3 consistently activate oxidative bursts and chemotaxis in mast cells in the form of an autocrine loop. Both mast cells and macrophages have been reported express CXCR3 (76). In the study of gallbladder cancer with ErbB pathway mutation, the interaction between CXCL10 and CXCR3 secreted by macrophages is induced, thus promoting the progression of gallbladder cancer (77). In addition, ligand-receptor pairing analysis exhibited stronger interaction of CXCL10-CXCR3 in CD4+ T and Treg cells of patients with ErbB pathway mutations than that in patients with non-ErbB pathway mutations. They found that immuno-suppressive macrophages increased expression of CXCL10 that binds to its receptor CXCR3 on Treg cells, eliciting tumor immune resistance in ErbB-mutation gallbladder cancer.

### CXCR3 expression on DC cells

In the study of blocking the immune checkpoint of hepatocellular carcinoma, Chuah et al. believed that the recruitment of CXCR3+ T and conventional dendritic cells (cDCs) could enhance the efficacy of anti-PD-1 and reduce the occurrence of adverse reactions (78). Stimulation by NK-DC cross talk cultures exposed to complement-opsonized-HIV led to the upregulation of CD38, CXCR3, and CCR4 on T cells (79). CXCR3 and CCR7 were highly expressed on plasmacytoid dendritic cells (pDCs), CXCR4 was expressed on both pDC and myeloid dendritic cells (80). CXCR3 in circulating pDCs is highly expressed in the inflammatory infiltration around the blood vessels of diabetic rats. The expression of CXCR3 correlated inversely with the frequency of pDCs in peripheral blood. CXCR3, CXCR4, and CCR7 might participate in the recruitment of DC from the peripheral blood of dermatomyositis patients to muscle tissue through different mechanisms. Gardner and their team recently shown that TIM-3 blockade promotes expression of CXCR3 chemokine ligands by tumor cDCs (81, 82). More than 60% of tumor-infiltrating CD8+ T cells expressed CXCR3 on their surface. CXCR3 at lower levels on CD4+ T cells and a small population of cDC1s, with no change in expression observed following treatment with αTIM-3/PTX. CXCR3 chemokine expression by tumor cells, macrophages, and cDCs plays essential roles in different systems (60, 83).

### CXCR3 expression on fibroblasts

Fibroblasts in the immunosuppressive microenvironment can prevent T cells to reach tumor cancer nests. Gorchs et al. found that CXCR3 ligands are associated with an increase in the number of T cells in tumor-rich areas, and cancer-associated fibroblasts (CAFs) down-regulate CXCR3 expression on T cells (84, 85). CXCR3 ligands can mediate T cell trafficking, and CAFs can prevent T cells from contacting malignant cells. CXCL9, CXCL10, CXCL11 and CCL8 levels were positive correlated with a higher ratio between CD8+ T cells in tumor areas and the total stroma, they might affect the spatial distribution of CD8+ T cells in human pancreatic tumor tissues. Conversely, CXCL10 levels showed to correlate with cancer cell invasiveness in other studies. It is also associated with gemcitabine resistance and worse survival of patients (86, 87).

## Mechanism and role of CXCL9,10,11/CXCR3 pathway in cancer

CXCL9, 10, and 11 are mainly secreted by monocytes, endothelial cells, fibroblasts, and tumor cells. CXCL9, 10, and 11 and CXCR3 pathways have two modes of action: immune-activated secretory signaling and tumor cell-derived proliferative metastatic signaling (30). The CXCL9,10,11/CXCR3 signaling pathways in the TME mainly facilitate the chemotactic movement of CXCR3-activated immune cells to the tumor site for anti-tumor immunity (88). Secretory signals of immune activation mainly affect the migration, differentiation, and activation of immune cells. In tumor cell-derived signaling, tumor-derived ligands render tumor cells metastatic mainly through CXCR3-A, and tumor-derived chemokines promote the recruitment of Th2 cells, regulatory T cells (Tregs), and myeloid-derived suppressor cells (MDSCs), producing tumor-promoting effects. CXCL9 and CXCL10 are critical components of many immune responses, activating NK and Th1 cells by binding to CXCR3 and inducing their entry into the sites of inflammation. It has been demonstrated (89) that high expression of CXCL9 in human early stage non-small cell lung cancer, recombinant human cytokine CXCL9 (rhCXCL9), or ligand transfer of CXCL9 can inhibit tumor-derived angiogenesis and suppress the growth and metastasis of tumor cells. Meanwhile, one investigator (90) found in a mouse model that CXCL9 expressed by tumor cells suppressed local tumor growth and metastasis by recruiting host NK cells and a large number of CXCR3+CD4+ and CXCR3+CD8+ host T cells. This provides evidence that high expression of CXCL9 and CXCL10 in the intra-tumor environment of the body is significantly correlated with a high density of CD8+ T cells and that increased selective expression of CXCL9 and CXCL10 in Treg cell-depleted tumors may serve as a potential immunotherapy. CXCL9 and the 10,11/CXCR3 axis in the TME are shown in **Figure 1B**.

However, in hepatocellular carcinoma studies, CXCL9 binds to the receptor subtype CXCR3 and activates the rhCXCL9-induced p-ERK1/2-MMP2/MMP9 pathway, enhancing the migration and invasion of CD133+ hepatocellular carcinoma (91). Ejaeidi et al. (92) found significantly elevated expression levels of CXCL9, CXCL10, and CXCL11 in the serum of patients with metastatic breast cancer. These three chemokines play essential roles in the development of breast cancer by activating survival proteins, β-strand proteins, mitogen-activated protein kinase phosphatase 1 (MKP-1), and matrix metalloproteinase 1 (MMP-1). In addition, inhibition of CXCL9 expression promotes intracellular actin polymerization, cell adhesion, and cell survival; increases the intracellular calcium concentration; and induces the migration of melanoma cells (93). In colorectal cancer studies, ligands that recruit Th1 cells, CTL, NK cells, NKT cells, and M1 macrophages to the tumor site could be used as effective anti-tumor treatment options. Treatment with plasmid vectors carrying CXCL9 in combination with cisplatin can control the progression of colon and lung cancers and enhance CTL activation (94). The relationship between the CXCL9,10,11/CXCR3 pathways and PD-1/PD-L1 is a vital area of research, and combining this pathway with other immunotherapies improves the efficacy of tumor immunotherapy by enhancing the inhibition of tumor progression through multiple mechanisms.

## The role of CXCR3 and its ligands in cancer

CXCR3 has been detected in many malignant cell lines and is correlated with the prognosis of patients with melanoma, colon cancer, and breast cancers (95–97). Among them, high CXCR3 expression in melanoma, colon, and breast cancers is associated with more malignant and aggressive tumors. In contrast, in renal cell carcinoma and chronic lymphocytic leukemia (CLL), low CXCR3 expression implies a shorter survival time for patients (98, 99). Combined studies have shown that CXCR3 and its ligands have a bidirectional regulatory role in the biological behavior of tumors. On the one hand, CXCR3 and its ligands can inhibit tumor growth by activating immune effector cells; on the other hand, CXCR3 exhibits a role in promoting tumor growth and metastasis in some tumors.

The anti-tumor effect of CXCR3 has been demonstrated by its ability to activate the autoimmune system response to achieve anti-tumor growth. The earliest study was in 1993, when Luster et al. (100) found that the CXCL10/CXCR3 pathway could exert anti-tumor effects by recruiting lymphocytes to the tumor tissue. With further research on the critical role of the tumor environment in the immune system, it was found that CXCR3 and its ligands may exert anti-tumor effects by mediating chemotaxis and inhibiting angiogenesis. CXCR3 is also expressed in some tumor cells and vascular endothelium. It has been shown that CD4+ Th1 cells and effector CD8+ T lymphocytes, which proliferate and differentiate after initial T lymphocyte activation, can specifically express CXCR3 (31). Under the chemotactic effect of CXCL9, CXCL10, and CXCL11, activated Th1 cells reach the tumor site to exert phagocytosis and upregulate CXCL9, CXCL10, and CXCL11 secretion, thus recruiting more T lymphocytes to participate in the immune response at the lesion site. CXCL10 activates and chemoattracts NK cells and CD8+ T lymphocytes to reach the site of tumors and exerts a pernicious effect on tumor cells. CXCL10 is highly expressed in mouse models of plasmacytoma and mammary adenoma, while it has low expression in mouse models without thymus; therefore, the anti-tumor effect of the CXCL10/CXCR3 pathway is thought to be T lymphocyte-dependent (100). Regarding mouse malignant gliomas, when GL261 cancer cells were transfected with CXCL10 and subcutaneously transplanted into mice, tumor progression was significantly delayed, suggesting that the CXCL10/CXCR3 pathway can effectively inhibit tumor progression. CXCR3B is a typical ELR-CXC chemokine primarily expressed in vascular endothelial cells and has an inhibitory effect on angiogenesis. In breast cancer, CXCR3B exerts its role in inhibiting angiogenesis by reducing DNA synthesis and activating specific signal transduction pathways (101).

In contrast, CXCR3 promotes tumor growth and metastasis while inhibiting tumor growth. Several studies have demonstrated the anti-tumor effects of CXCR3 and its ligands. Recent studies have revealed that the chemokine receptor CXCR3 and its ligands are not only expressed in immune cells but are also highly expressed in a variety of tumor cells, and its different variants have very different biological effects on tumors. In a study of gliomas, Wang (102) concluded that DC-induced CTL synergistically inhibited glioma growth while suppressing tumor angiogenesis and enhancing cytotoxicity, thereby increasing the number of brain-infiltrating lymphocytes (BILs) and prolonging the residence time of CTL in the tumor. Ma et al. (17) found that CXCR3 expression may correlate with prognosis in breast, melanoma, colorectal, and renal cancer cells. Another study reported two isoforms of CXCR3, CXCR3A and CXCR3B, which have opposite biological functions (103). In colorectal cancer cell lines, CXCR3B mRNA was less expressed in the colorectal cancer cell line LOVO, and the migration ability of the cancer cell line LOVO decreased after overexpression of CXCR3B, suggesting that CXCR3B can inhibit the migration of cancer cells. Many studies have confirmed that CXCR3 is expressed and mediates cellular functions in malignant cells such as the breast (104–106). Breast cancer cell lines represent CXCR3-A and CXCR3-B, and these two receptor variants play different roles in endothelial cells (105). Li et al. (19) found that the expression level of CXCR3A is much higher than that of CXCR3B in primary invasive ductal carcinoma tissues of the breast, suggesting that the CXCL10/CXCR3 pathway plays an essential role in the development and progression of breast cancer. Chen et al. (107) found that CXCR3 mRNA and protein levels were significantly higher in human gastric cancer tissues and cell lines than in paracancerous tissues by protein blotting assay, suggesting that CXCR3 plays an essential role in the development and progression of gastric cancer.

CXCR3 and its ligands also play crucial roles in tumor metastasis. CXCR3 is widely expressed in melanoma cell lines, and CXCL9 stimulation promotes melanoma cell migration. Highly metastatic B16F10 and low-metastatic B16F1 cell lines have shown comparable levels of CXCR3 (108). Silencing CXCR3 ligand expression in B16F10 cells suppressed the development of melanoma lymph node metastasis, while CXCR3 expression had little effect on locally implanted tumors. One study induced CXCL9 and CXCL10 by adjuvant injection into the lymph nodes of melanoma patients to promote homing of tumor cells to inflamed lymph nodes; CXCR3 was detected by immunohistochemistry in approximately one-third of tumor specimens, and its expression was positively correlated with tumor size and the presence of distant metastases (95). These studies support the hypothesis that CXCR3 mediates melanoma metastasis to draining lymph nodes.

CXCR3 ligands have been suggested to play an essential role in IL-7/IL-7Rα-Fc-mediated anti-tumor activity in lung cancer studies (109). Neutralizing CXCL9, CXCL10, or IFN-γ reduces CXCR3-expressing activated T cells infiltrating tumors and abrogates IL-7/IL-7rα-Fc-mediated tumor growth inhibition. Also, in a study on pulmonary metastasis from osteosarcoma (110), it was concluded that targeting CXCR3 could specifically inhibit tumor metastasis without adversely affecting the anti-tumor host response. Barash et al. (111) suggested that acyl heparanase induction is associated with reduced CXCL10 levels, suggesting that this chemokine plays a tumor-suppressive role in myeloma.

In a study on breast cancer, Bronger et al. (15) found that the median overall survival and progression-free survival of breast cancer patients with high CXCR3 expression were lower than those of patients with common CXCR3 expression. Ma et al. (97) examined CXCR3 expression levels in the cytoplasm and cytosol of 75 cancer cells from patients diagnosed with stage I/II breast cancer and found that patients with high CXCR3 expression had shorter overall survival and that CXCR3 expression levels were positively correlated with tumor size, metastasis, or the number of involved lymph nodes, suggesting that high CXCR3 expression is closely associated with poor prognosis in patients with early stage breast cancer. The suppressed anti-tumor effect of CXCR3 might be related to the deficiency of functional NK cells and IFN-γ.

In ovarian cancer studies in women, Windmulle et al. (14) found that CXCR3 was significantly highly expressed in both the primary site of ovarian cancer and in abdominal metastatic lesions. In an *in vitro* assay, it was found that the production of malignant ascites in ovarian cancer could be inhibited by anti-CXCR3 monoclonal antibodies, thereby inhibiting tumor cell metastasis, suggesting that CXCR3 could be an essential indicator for evaluating the prognosis of ovarian cancer patients.

At the same time, regarding the digestive system, there is a considerable number of esophageal, gastric, and colorectal cancer patients. Yang et al. (17) found that in gastric cancer tissues, CXCR3A may contribute to the metastasis of cancer cells by upregulating the expression of matrix metalloproteinase 13 (MMP-13) and IL-16 and inducing the activation of the ERA1/2 pathway. A significant reduction in MMP-13 and IL-6 secretion was observed after the knockdown of CXCR3A. Murakami et al. (112) found that high CXCR3 expression promoted lymph node metastasis in colorectal cancer, and lymph node and organ (mainly the liver and lung) metastasis occurred significantly less in colorectal cancer tissue samples after CXCR3 knockdown. Colon cancer cell lines express all known CXCR3 variants (96, 113). In addition, CXCL10 leads to cell migration, calcium mobilization, and activation of ERK1/2 and AKT, accompanied by the induction of MMP2 and MMP9. The probability of metastasis to the lung or liver in colorectal cancer is not affected by altered CXCR3 expression levels. Altering CXCR3 levels did not affect the size of primary tumors. Examination of CXCR3 in primary colon cancer tissues revealed high CXCR3 expression in approximately one-third of the tumors, whereas half were positive for CXCR4 expression. Patients with CXCR3-positive tumors were significantly more likely to develop lymph node metastasis. This suggests that CXCR3 is an independent risk factor for a poor prognosis. Similar to colorectal cancer expression, it has been indicated that CXCR3 and its ligand axis can promote cancer cell proliferation and metastasis in esophageal cancer (114).

In the urology study, Vollmer and his team (115) found that in treating patients with muscle-invasive bladder cancer, the intratumoral CXCR3 chemokine system (ligands and receptor splice variants) was identified as a critical component for tumor eradication upon neoadjuvant chemotherapy. Their data revealed the stimulatory activity of the CXCR3ALt-CXCL11 chemokine system on CD8+T cells and predicted the chemotherapy response in muscle-invasive bladder cancer. This may provide an immunotherapy option for the targeted activation of intratumoral stem cell-like T cells in solid tumors. Utsumi et al. (13) found that renal cancer tissues expressed six times more CXCR3 than normal kidney tissues, whereas the expression of CXCL9 and CXCL11, the ligands of CXCR3, was 12 and 8 times higher than that in normal kidney tissues, respectively. CXCL10 was 22 times higher than that in normal kidney tissues, suggesting that the CXCR3/CXCL10 biological axis may play an important role. In patients with renal cell carcinoma, 5-year disease-free survival was significantly better in patients with low CXCR3-expressing tumors (99). Wu et al. (116) examined two major splice variants of CXCR3 in prostate cancer tissue samples. They found that CXCR3 mRNA and protein levels were higher in prostate cancer tissue specimens than in normal tissues, and the levels of CXCR3A mRNA were higher than those of CXCR3B mRNA. This may be because CXCR3 activation of the PLC-β/Ca^2+^ signaling pathway reduces the inward flow of intracellular calcium ions, decreases the adhesion of cells to the substrate through the activation of μ-calpain, and contributes to tumor cell migration. Nagaya et al. concluded (117) that in the metastatic prostate, significantly higher expression of ELR+ CXC chemokines/receptors and significantly lower expression of ELR-CXC chemokines/receptors were observed in bone metastases relative to lymph node metastases and that co-expression of CXCL10/CXCR3 was associated with postoperative recurrence. In addition, Utsumi et al. (13) confirmed in their study that the higher the CXCR3A/CXCR3B ratio in kidney cancer tissues, the higher the chance of tumor metastasis. CXCR3 is also expressed in other malignancies; however, the relationship between high CXCR3 expression and poor prognosis does not apply to all malignancies. The significance of CXCR3 and its ligand pathway in the immune environment of common tumors is summarized in **Table 1**.

**Table 1:** Significance of CXCR3 and its ligand pathway in different tumor immune environments.

Tumor Type	Ligand	Receptors	Meaning	Mechanism of action	Role in the TME	Results	Ref.
Colorectal Cancer	CXCL9/10	CXCR3	Promoting lymph node metastasis	CXCL10 and CXCL12 induce directional migration of cancer cells. Activated CXCR3 has a synergistic effect on CXCR4 to promote tumor metastasis indirectly, so CXCR3 positivity suggests a poor prognosis.	Induction of CTLs	Decrease in tumor microvessel density and increase in apoptosis	(71)
			Promotes tumor growth and metastasis	CXCR3 has low expression in non-tumor tissues, and low expression of CXCR3B can inhibit tumor cell spreading, so high CXCR3B expression suggests good prognosis.			(93)
			Accelerated apoptosis	Combination treatment with CXCL9-containing plasmid vector and cisplatin on the density of microvessels in the microenvironment of colorectal cancer tumor cells, which in turn can accelerate apoptosis of tumor cells.			(53)
Stomach Cancer	CXCL10	CXCR3	Promotes tumor growth	Among the heterodimers of CXCR3, CXCL10 promotes the invasion and migration of gastric cancer cells through CXCR3A, but not the growth and metastasis of gastric cancer cells through CXCR3B. High expression of CXCR3B suggests a good prognosis.	Induction of CTLs	Increased tumor angiogenesis	(16)
Esophageal Cancer	CXCL9/10/11	CXCR3	Promotes proliferation and metastasis of cancer cells	The chemokine receptor axis is prominent in esophageal adenocarcinoma, with a fold change of up to 9.5. Its immune regulation is mainly driven by the chemokine CXCR3/CCR5 axis and cytotoxic effector mechanisms essential for T cell activation and differentiation to promote cancer cell proliferation and metastasis.	Tumor-associated fibroblasts and elevated IL-12	Increased angiogenesis in the tumor microenvironment, mitosis of cells	(73)
Breast Cancer	CXCL9/10	CXCR3	Promotion of lymph node and lung metastasis	CXCR3 was highly expressed in the cytoplasm and cytosol of cancer cells in patients with early breast cancer. This study showed that patients with high CXCR3 expression had shorter overall survival, and the expression level of CXCR3 was positively correlated with tumor size and the number of lymph nodes involved in metastasis, so high CXCR3 expression suggested a poorer prognosis for patients with early breast pain.	Induction of Th1 and CTLs in the peripheral blood	Inhibition of lung metastasis, but not local cancer growth	(56)
			Promotes tumor growth and metastasis	(1) The median overall survival and progression-free survival of breast cancer patients with high CXCR3 expression are lower than those with lower CXCR3 expression, so CXCR3 positivity suggests a poorer prognosis.			(15)
				(2) CXCR3B plays an anti-proliferative role by inhibiting the ERK1/2 signaling pathway and p38 kinase, so high expression of CXCR3B suggests good prognosis, and increased expression of CXCR3A means poor prognosis.			(19)
Prostate Cancer	CXCL10	CXCR3	Promotes tumor growth and metastasis	CXCR3B inhibits the cAMP/PKA signaling pathway to reduce cell migration, and CXCR3 activates the PLC-B/Ca^2+^ pathway to promote tumor cell dissemination and metastasis, so high expression of CXCR3B suggests a good prognosis.	Induction of CTLs	Increases vascular density in the tumor microenvironment and promotes tumor growth and lymph node metastasis	(75)
			Postoperative recurrence and lymph node metastasis	In the metastatic prostate, significantly higher expression of ELR+CXC chemokines/receptors and significantly lower expression of ELR-CXC chemokines/receptors were observed in bone metastases relative to lymph node metastases, and co-expression of CXCL10/CXCR3 was associated with postoperative recurrence.			(76)
Kidney Cancer	CXCL9/10/11	CXCR3	Promotes distant metastasis	CXCR3 mediates the directional movement of kidney cancer cells, which promotes the metastasis of kidney cancer cells, so CXCR3 positivity indicates a poor prognosis.	Induction of mononuclear cells	Reduction in tumor growth and angiogenesis	(13)
Ovarian Cancer	CXCL9/10	CXCR3	Promotion of malignant ascites production	CXCR3B inhibits the cAMP/PKA signaling pathway to reduce cell migration, and CXCR3 activates the PLC-B/Ca^2+^ pathway to promote tumor cell dissemination and metastasis, so high expression of CXCR3B suggests a good prognosis.	Induction of CTLs	–	(14)
Lung Cancer	CXCL9/10	CXCR3	Promotes tumor growth and metastasis	CXCR3 ligands play an essential role in IL-7/IL-7Rα-Fc-mediated anti-tumor activity. Neutralizing CXCL9, CXCL10, or IFNγ reduces CXCR3-expressing activated T cell infiltration into tumors and abrogates IL-7/IL-7rα-Fc-mediated tumor growth inhibition.	Induction of CTLs	Decrease in tumor microvessel density and increase in apoptosis	(68)
Glioma	CXCL10	CXCR3	Inhibits tumor growth	Interferon (IFN)-γ-inducible protein (IP)-10 (IP-10) is a potent angiogenesis inhibitor that recruits CXCR3+ T cells, including CD8+ T cells. In the presence of IP10-scFv and EGFRvIII peptide pulses, DC-induced CTL synergistically inhibits glioma growth while suppressing tumor angiogenesis and enhancing cytotoxicity, thereby increasing the number of brain-infiltrating lymphocytes (BILs) and prolonging CTL residence in the tumor.	Induction of CTLs	Reduction in tumor growth and improved prognosis	(61)
Osteosarcoma	CXCL9/10/11	CXCR3	Inhibit tumor growth and metastasis	CXCR3 and its ligands interfere with the initial spread of osteosarcoma cells to the lung and stimulate the growth and expansion of metastatic foci at a later stage. Targeting CXCR3 specifically inhibits tumor metastasis without adversely affecting the anti-tumor host response.	To mediate TAM targeted therapy	Inhibition of lung metastasis (*in vivo*), and cancer cell growth (*in vitro*)	(69)
Myeloma	CXCL10	CXCR3	Inhibits tumor growth	Acylheparanase induction was associated with reduced CXCL10 levels, suggesting that this chemokine plays a tumor suppressive role in myeloma. Indeed, recombinant CXCL10 attenuated the proliferation of CAG, U266, and RPMI-8266 myeloma cells.	Induction of CTLs and NK cells	Reduction in tumor growth	(70)

## CXCR3 and its ligand in therapy

An in-depth study of the body’s immunity and internal environment has shown that the application of immune activators plays an essential role in the treatment of cancer. Currently, immunotherapy for CXCR3 mainly includes antagonists and agonists, among which inhibitors include widely used inhibitors (AMG487 and TAK-779). The agonist PS372424 is a specific agonist of CXCR3. The effects of these drugs were verified in animal models. As mentioned above, CXCR3 plays a dual role in affecting tumor progression and the microenvironment, and its effects vary when immunotherapy is used against different tumors. CXCR3 has been associated with tumor growth in several other diseases such as rheumatoid arthritis, atherosclerosis, and inflammatory skin diseases (118, 119). A minor molecular weight antagonist of CXCR3, AMG487, was found to inhibit tumor metastasis in a mouse breast cancer model (120). In addition, CXCR3 antagonism by AMG487 inhibited lung metastasis in a mouse osteosarcoma model and metastatic colon cancer model (110, 121). Antisense RNA targeting of CXCR3 during cancer treatment with immunosuppressive agents inhibits the metastasis of B16F10 melanoma to lymph nodes but does not affect the possibility of tumor metastasis to the lung (108). The administration of neutralizing antibodies against the ligands CXCL9 and CXCL10 produced a similar protective effect. AMG487, a minor molecular weight CXCR3 antagonist, may inhibit the possible spontaneous metastasis of breast tumors to the lungs *via* a systemic route of administration (120). AMG487 has been developed as a potential therapeutic agent for the treatment of inflammatory diseases. Although it did not show efficacy in clinical trials for the treatment of psoriasis, the drug was well tolerated. Thus, it may be a promising candidate for evaluation in clinical cancer trials. CXCR3 short-stranded RNA expressed in breast tumor cells effectively controls breast tumor metastasis. Whether genetic or pharmacological approaches inhibit CXCR3, its ability to affect breast cancer metastasis depends on the presence of normal NK cells. Through a controlled study in mice, Saleh et al. (122) found that AMG487 exerts anti-arthritic effects by significantly downregulating inflammatory B-lymphocyte signaling pathways. This suggests that AMG487 may serve as a novel therapeutic agent for inflammatory and autoimmune diseases. Another antagonist, TAK-779, which has affinity for CXCR3, CCR5, and CCR2b, has been studied in a mouse model of rheumatoid arthritis (123). Selective targeting of CCR5/CCL5 signaling by TAK-779 in a mouse pancreatic cancer model may represent a novel immunomodulatory strategy for cancer treatment (124). Currently, CXCR3 agonists (PS372424) are mainly used in preclinical studies. Intravenous treatment with PS372424 prevented the inflammatory migration of activated human T cells toward murine air pouches filled with chemokines or synovial fluid from patients with active rheumatoid arthritis (125). Further studies are required to confirm the application of these agonists.

However, because of the differential expression of CXCR3 in different cancer diseases, its special function in the immune system makes it a potential target for immunotherapy. For example, in clinical colorectal cancer patients, CXCR3 expression levels are significantly higher in lymph nodes (LN) and metastases in the liver compared with those in primary tumors. CXCR3 activation promotes cancer migration and growth *in vitro* and *in vivo*, and both responses can be inhibited and eliminated by a CXCR3 antagonist (AMG487). CXCR3 is detected in approximately one-third of early colorectal cancer tumor specimens, and patients with CXCR3-positive tumors tend to have lymph node metastases more often, suggesting that CXCR3 is an independent risk factor for poor prognosis (126). In colorectal tumors, CXCR3 is mainly expressed in the cytoplasm and cell membrane of tumor cells. The expression level of CXCR3 was significantly correlated with clinicopathological factors such as tumor differentiation, lymph node metastasis, distant metastasis, and Dukes’ stage (127). However, there was no significant correlation between sex, age, and tumor location. Wu et al. (128) concluded that CXCR3 mRNA and protein expression levels were significantly higher in colorectal tissues than in the corresponding non-cancerous tissues. The overall survival was significantly lower in CXCR3-expressing patients. Fulton (126) verified in an experimental model of colorectal cancer animal metastasis that inhibition of CXCR3 expression in tumor-bearing mice reduced the probability of lung metastasis of tumors. Although CXCR3 plays a crucial role in lymph node metastasis, its role in colon cancer metastasis to the liver and lungs has not been fully elucidated. The importance of CXCR3 expression on Tregs, Teffs, and macrophages in the induction and progression of colorectal has been demonstrated in previous studies. In conclusion, numerous studies have shown that CXCR3 promotes metastasis of colorectal cancer to lymph nodes (96). Current immunotherapies targeting T-lymphocyte checkpoints, such as programmed cell death (PD-1), are among the most promising and exciting new approaches to cancer treatment.

Prior work has suggested that CXCR3 is mainly expressed by stem-like PD­1loCD8+ T cells, whereas CCR5 is elevated on terminally exhausted PD-1hiCD8+ T cells (36, 63, 129). In addition, CXCR3 has been identified as a limiting factor for immune checkpoint blockade efficacy in preclinical models and was implicated in CD8+ T cell trafficking and positioning within tumors (22, 36, 60, 61). And the relationship between CXCR3 and immune checkpoint inhibitors has been shown. Li et al. demonstrated that overexpression of Glycoprotein - A repetitions predominant in human tumors is associated with poor clinical response to TME and ICB (130). His team use Glycoprotein - A repetitions predominant antibody treatment to reduce the typical TGF signaling pathway in tumor-infiltrating immune cells, prevent T cell exhaustion, and promote CD8+ T cell migration to TME in a CXCR3-dependent manner to enhance anti-tumor effects. In a PD-1/PD-L1 immunotherapy study, Van Braeckel-Budimir et al. (59)treated Mc38-bearing mice with triple combinatorial therapy (including anti-OX-40, anti-4-1BB, anti-PD-1 or anti-PD-L1) together with neutralizing antibodies against CXCR3 or control antibodies. Consistent with the role of CXCR3 in intratumoral T cell trafficking, CXCR3 neutralization greatly impaired the accumulation of total and CD8+ T cells in the TME induced by triple combinatorial therapy. It is believed that the efficacy of triple combinatorial therapy largely depends on CXCR3-mediated intratumoral stem cell recruitment and CD8+ T cell metastasis. The relationship between the CXCL9,10,11-CXCR3 pathway and PD-1/PD-L1 was found to be an essential area of research during the study of immunotherapy in colorectal cancer (131), and combining this pathway with other immunotherapies improves the efficacy of tumor immunotherapy by enhancing the inhibitory effect on tumor development through multiple mechanisms. Using an experimental mouse hormonal tumor group, Chow et al. (36) showed that the successful application of anti-PD-1 immunotherapy requires the expression of CXCR3 by CD8+ T lymphocytes and the production of CXCL9 by CD103+ DCs. In anti-PD-1-treated mice, CXCR3 and its ligands are essential in generating CD8+ T lymphocyte responses, and the production of CXCL9 promotes anti-PD-1-induced anti-tumor responses, suggesting that CXCR3 facilitates the interaction between DCs and T lymphocytes in the TME. In an adoptive T cell transfer study, Pilipow (132) found that CD8+ T cells treated with N-acetylcysteine showed the presence of IL-2 and IL-12. Compared with cells activated with beads conjugated with anti-CD3/CD28 antibody, the surface expression of CD95 was reduced. However, the expression of CD45RA, CD27, and CXCR3 increased, indicating that stem cell memory T cells were genuinely differentiated and thus exhibited stronger anti-tumor immunity. The characteristics and roles of the CXCR3 chemokine system make it a potential target for immunotherapy and offer new hope for tumor treatment.

The in-depth study of CXCR3 has provided a more profound understanding of diseases involving CXCR3 and new treatment avenues to explore. In general, most therapeutic approaches based on the CXCR3 axis can be divided into two types, one is carried out by inhibiting CXCR3 activation, and the other is inhibiting angiogenesis by exogenous CXCR3 ligands or their derived peptides. CXCR3 activation might also promote an effective immune response. Based on the available evidence, many experimental approaches have been studied to treat tumors, including different cancer models, combinations with other therapies, and drug delivery strategies ranging from intratumoral injection of chemokines to viral vector therapy (133). The related role of the CXCR3 ligand is also being explored, and changes in CXCR3 ligand recognition *in vivo* can fine-tune the effect of CXCR3 anti-tumor activity. Some studies have confirmed that this function of the CXCR3 ligand is derived from the carboxy-terminal peptide of platelet factor (134, 135). It is possible to combine CXCL10 and CXCL10 chimeric chemokine CXCL11 or other ligands to achieve an effect that can increase cell-to-cell permeability. However, Karin (27) suggested that CXCL10 and CXCL9 may differ from other chemokines in their ability to inhibit tumor growth and enhance anti-tumor immunity. It is believed that in various human cancers, low expression of CXCL10 at the tumor site is significantly associated with poor prognosis. The differences between CXCL9 and CXCL10 in their biological functions, possibly through biased signaling, and their possible relevance to cancer immunotherapy have been discussed (136). CXCR3 and its ligands provide an accessible indicator for diagnosis, prognosis, and therapy responsiveness in many diseases. In addition to its role in tumors, CXCR3 also exists in almost all diseases involving immunity. For example, in both Crohn’s disease and rheumatoid arthritis, high plasma levels of CXCL4 have been shown to predict non-responsiveness to anti-TNF-α antibody (infliximab) therapy (137, 138).

Tumor development depends not only on the tumor cells themselves, but also on the TME in which the tumor cells are located. Chemokines are small molecule proteins that mediate the directional movement of cells. They are essential for inducing leukocyte chemotaxis, promoting differentiation and proliferation, and inducing tissue extravasation. In the TME, chemokines undergo complex and dynamic interactions with tumor cells by binding to their corresponding receptors and recruiting immune cells to infiltrate the tumor tissue. At the same time, chemokines can also bind to receptor-containing tumor cells to promote tumor growth, metastasis, and invasion. In the TME, both CXCR3-A and CXCR3-B can attach to the ligands CXCL9/10/11, mediating opposite biological functions. CXCR3-A induces cell proliferation and target chemotaxis. Its signaling mechanism activates Gi and Gq proteins to trigger downstream signaling pathways, including intracellular Ca^2+^ release, ERK1/2, and other effects (139, 140). CXCR3-B binding to ligands inhibits cell migration, proliferation, and apoptosis, and the mechanism underlying the opposite cellular response remains to be further investigated. Tumorigenesis is a complex and dynamic process involving various cellular and noncellular factors in the TME. In addition to tumor cells, the TME also includes tumor-associated fibroblasts, tumor vascular endothelial cells, immune and inflammatory cells, myeloid-derived cells, and the extracellular matrix (141), which are closely associated with tumor cell interactions and their tumorigenesis, development, and drug resistance. In the TME, CXCL9, CXCL10, and CXCL11 are mainly secreted by monocytes, endothelial cells, fibroblasts, and tumor cells and mediate the migration, differentiation, and activation of immune cells by recruiting various immune cells (CTL, NK cells, macrophages, etc.) in an autocrine and paracrine manner (31, 142). The immunoreactivity of tumors depends on the type of immune cell infiltration and is divided into “hot tumors,” which are more immunoreactive, and “cold tumors,” which are less immunoreactive (30). All three ligands are effective against activated Th1, CTL, and NK cells (143). CXCL9 induces NK cells into tumors, and CXCL9-rich cells are less tumorigenic than CXCL9-deficient cells in the mouse liver (144). However, Tregs can also be attracted to the TME *via* a chemokine gradient (CXCR3-CXCL9/10/11) that activates Tregs and suppresses anti-tumor immune responses; thus, high Treg infiltration is associated with poor survival in various tumor types (145). Liu et al. (146) found that the upregulation of CXCL10 and IL-6 expression triggers M1 macrophage polarization, promotes dendritic cell migration and maturation, mediates the migration of CD4+ T cells and their differentiation into Th1 and CD4+ CTL, and reduces the accumulation of myeloid-derived suppressor cells. In the TME, chemokines act at three levels: inflammation, immunity, and vascular/nonvascular tumor mesenchyme. Thus, their role in tumorigenesis is complex because of their multi-target properties and many feedback loops. CXCR3 is present in tumor and immune cells, and its two variants, CXCR3-A and CXCR3-B, have different molecular characteristics and can mediate biological effects with opposite results. Blocking CXCR3-A in tumor cells may be a novel anti-tumor strategy to prevent tumor cell invasion and metastasis. In addition, other players in the TME, including growth factors, inflammatory cytokines, and extracellular matrix enzymes, add to the complexity of CXCR3 and ultimately render the outcome unpredictable. Therefore, before using CXCR3 variants as potential anti-tumor targets, a better understanding of the biology of CXCR3 variants is needed to clarify their exact signaling pathways and cellular responses in the TME to lay a solid foundation for precise or combination tumor treatment. Autocrine and paracrine CXCR3 signaling pathways are shown in **Figure 1C, D**.

The above studies found that chemokine receptor function in the body not only guides the transport of tumor cells but also plays a vital role in immunotherapy. Although these findings are instructive, the ability of CXCR3+ T lymphocytes and NK cells to exert endogenous tumor control may be compromised if there is long-term systemic antagonism of CXCR3 in the body’s internal environment. In addition, the antiangiogenic properties of CXCR3 ligands may be negated by CXCR3 antagonism. Thus, elucidating a more profound role for CXCR3 requires a broader assessment of the impact of receptor targeting on potential anti-tumor immune effector cell functions.

## Conclusion and prospects

The chemokine receptor CXCR3 and its specific ligands play key roles in tumorigenesis, progression, and metastasis. The expression of CXCR3 and other chemokine receptors on malignant cells is well documented, and their biological behavior in tumor immunity is quite complex and influenced by a variety of factors, including the type of chemokine receptor splice variants, the type of cells in which they are expressed, and the TME. CXCR3 is expressed and secreted by tumor epithelial, inflammatory, and endothelial cells in the TME. Differential expression of CXCR3-A and CXCR3-B in different variants of CXCR3 leads to different physiological functions. At the same time, the role of CXCR3 ligands in treatment differs. The role of CXCR3 in tumor progression involves a complex signaling cascade. The differential expression of CXCR3 in many tumor tissues determines the inhibition or promotion of tumor growth. CXCR3 has been shown to play a significant role in many diseases in various clinical and prospective studies. However, the specific molecular biological mechanisms and related signaling pathways need to be investigated further. Several prospective studies have shown that reducing chemokine receptor function can significantly reduce metastatic tumor potential; however, the specific mechanisms behind this are largely unknown. The mechanism of action of CXCR3 in various tumors may be uncovered, and it is expected to become a new target for clinical tumor immunotherapy.

## Author contributions

XW designed this review and write the manuscript. BX, LY, and GC helped to design this review and write the manuscript. YZ, SW, HN, and PZ assisted in writing and editing the manuscript. BX, LY, GC, and PZ reviewed and revised the manuscript. All authors contributed to the article and approved the submitted version.

## Funding

This project was supported by National Cancer Center Climbing Fund (NCC201916B03), Provincial-ministerial Co-construction Project of Henan Province Science and Technology Key Point Tackling Plan (SBGJ202102064), and Henan Provincial Scientific and Technological Project (222102310363, 222102310677).

## Conflict of interest

The authors declare that the research was conducted in the absence of any commercial or financial relationships that could be construed as a potential conflict of interest.

## Publisher’s note

All claims expressed in this article are solely those of the authors and do not necessarily represent those of their affiliated organizations, or those of the publisher, the editors and the reviewers. Any product that may be evaluated in this article, or claim that may be made by its manufacturer, is not guaranteed or endorsed by the publisher.
